# mTOR pathway as a potential therapeutic target for cancer stem cells in canine mammary carcinoma

**DOI:** 10.3389/fonc.2023.1100602

**Published:** 2023-01-27

**Authors:** Masaki Michishita, Kazuhiko Ochiai, Rei Nakahira, Daigo Azakami, Yukino Machida, Tomokazu Nagashima, Takayuki Nakagawa, Toshiyuki Ishiwata

**Affiliations:** ^1^ Department of Veterinary Pathology, Faculty of Veterinary Science, Nippon Veterinary and Life Science University, Tokyo, Japan; ^2^ Research Center for Animal Life Science, Nippon Veterinary and Life Science University, Tokyo, Japan; ^3^ Department of Veterinary Hygiene, Faculty of Veterinary Science, Nippon Veterinary and Life Science University, Tokyo, Japan; ^4^ Laboratory of Veterinary Clinical Oncology, Faculty of Agriculture, Tokyo University of Agriculture and Technology, Tokyo, Japan; ^5^ Laboratory of Veterinary Surgery, Graduate School of Agricultural and Life Science, The University of Tokyo, Tokyo, Japan; ^6^ Division of Aging and Carcinogenesis, Research Team for Geriatric Pathology, Tokyo Metropolitan Institute of Gerontology, Tokyo, Japan

**Keywords:** cancer stem cells (CSC), dog, mammary adenocarcinoma, mTOR, sphere-formation assay

## Abstract

Mammary adenocarcinoma, the most common cancer in female dogs, often exhibits the lymph node and lung metastases and has a higher mortality rate. However, mammary adenocarcinoma has no established treatment, except early surgical excision. Canine mammary carcinoma has many common features with human mammary carcinoma, including clinical characteristics, heterogeneity, and genetic aberrations, making it an excellent spontaneous tumor model for human breast cancer. Diverse cancers comprised heterogeneous cell populations originating from cancer stem cells (CSCs) with self-renewal ability. Therefore, in addition to conventional therapy, therapeutic strategies targeting CSCs are essential for cancer eradication. The present study aimed to extract inhibitors of canine mammary CSCs that suppress their self-renewal ability. Sphere-formation assay, which evaluates self-renewal ability, was performed for the canine mammary cancer cell lines CTBp and CNMp. The spheres formed in this assay were used in inhibitor library screening, which identified various signaling pathways such as proteosome, stress inducer, and mammalian target of rapamycin (mTOR). The present study focused on the mTOR signaling pathway. Western blotting showed higher levels of phosphorylated mTOR in sphere-forming CTBp and CNMp cells than in adherent cells. Drug sensitivity examination using the mTOR inhibitors everolimus and temsirolimus revealed dose-dependent reductions in viability among both sphere-forming cells and adherent cells. Expression of phosphorylated mTOR in adherent and sphere-forming cells decreased by everolimus and temsirolimus treatment. In mice transplanted with CTBp-derived spheres, everolimus treatment significantly decreased tumor volume compared to control. These results reveal that the mTOR signaling pathway may be a potential to be a therapeutic target in both cancer cells and CSCs. Novel therapeutic strategies for canine mammary carcinoma are expected to benefit to human breast carcinoma as well.

## Introduction

Canine mammary tumors are the most common tumors in middle-aged and elderly female dogs (1). Canine mammary carcinoma accounts for approximately 50% of all mammary tumors and is associated with poor clinical behavior, including lymph node and lung metastases, and high mortality (1). Canine mammary cancer shares many common features with human breast cancer, including the age of onset, hormonal etiology, stage, tumor diversity, lymph node metastasis, and genetic abnormalities including breast cancer susceptibility gene 2 (BRCA), phosphatidylinositol-4,5-bisphosphate 3-kinase catalytic subunit alpha (PIK3CA), and TP53 mutations, and protein expression, including human epidermal growth factor receptor 2 (HER2), estrogen receptor, progesterone receptor, and p63 (2–6). Therefore, canine mammary cancer is considered a spontaneous model of human breast cancer. Sex hormones are closely related to mammary tumor development in dogs. Ovariectomy before the first estrous reduces the mammary tumor incidence by 99%, and ovariectomy before the second and third estrous reduces them by 92% and 74%, respectively (7). Surgical resection is the first choice for mammary tumor treatment in dogs. Adjuvant therapy is performed for inflammatory breast cancer for which quality of life improvement cannot be expected after surgical resection. Adjuvant therapy for canine mammary tumors includes chemotherapy, such as doxorubicin, cyclophosphamide, and docetaxel, and molecular-targeted therapy, such as toceranib, piroxicam, and firocoxib (8–12). Tamoxifen, which is used as estrogen therapy for human breast cancer, is not recommended in dogs due to adverse reactions, such as vulva edema, purulent discharge, pyometra, and conjunctivitis (13). Additionally, aglepristone, an anti-progestin drug, is used for labor induction and pyometra treatment. Aglepristone has not been practiced due to insufficient therapeutic outcome data, although it is expected to treat dogs with mammary cancers (5). Therefore, developing a novel treatment strategy in addition to conventional therapy is necessary because a treatment protocol, including adjuvant therapy for canine mammary cancer, has not yet been established. Human patients with breast cancer are treated with molecular-targeted drugs using monoclonal antibodies, tyrosine kinase inhibitors, cyclin-dependent kinase 4/6 inhibitors, antiangiogenic agents, and poly (ADP-ribose) polymerase inhibitors in addition to conventional chemotherapy (14). Molecular-targeted drugs were developed to directly act on molecular cancer cell abnormalities and selectively target various signaling pathways related to cancer cell proliferation, aggression, and apoptosis, and have yielded more successful results in cancer therapy (14). Canine mammary cancer has many similarities with human breast cancer, and molecular-targeted therapy for human breast cancer is expected to be beneficial in canine mammary cancer treatment.

Cancer stem cells (CSCs) or tumor-initiating cells are a subpopulation of cancer cells and play an important role in cancer development, recurrence, and metastasis. CSCs have self-renewal and differentiation capacities, higher tumorigenicity in nude mice, and radiotherapy and chemotherapy resistance (15, 16). Therefore, CSCs-targeted therapies are essential for cancer eradication. CSCs can be enriched by various techniques, such as surface antigen analysis, side population analysis, aldeflour assay, and sphere-formation assay, in humans and dogs (17, 18). Sphere-formation assay is an excellent method to efficiently enrich cell populations with self-renewal ability. Canine CSCs have been identified in various cancers, such as mammary adenocarcinoma, hepatocellular carcinoma, pulmonary adenocarcinoma, rhabdomyosarcoma, and melanoma since the existence of CSCs was first reported in osteosarcoma in 2007 (19–27). In veterinary medicine, CSC studies for mammary cancer are most advanced in dogs. Sphere-forming cells derived from mammary adenocarcinoma lines have higher stem cell-related gene expression and higher tumorigenicity in immunodeficient mice compared to adherent cells (20). Additionally, sphere-formation assay is used for *in vitro* sensitivity assay of anticancer drugs, such as doxorubicin and carboplatin, as well as small-molecule inhibitors targeting cyclooxygenase-2, and CSCs exhibit lower sensitivity than non-cancer stem cells (20, 28, 29). Therefore, sphere-formation assay is not only useful for elucidating the pathogenesis of mammary cancer, which is as diverse as in humans, but also for searching for inhibitors and molecular-targeted inhibitors that suppress self-renewal ability. High-throughput screening in humans, using sphere-formation assay, is conducted for various cancers, such as breast and lung cancers, for inhibitors that suppress the self-renewal ability of CSCs (30–32). High-throughput screening of canine mammary CSCs has not been reported although a few agents targeting canine mammary CSCs have been identified so far. Therefore, the present study used a molecular-targeted inhibitor library to search for drugs that suppress the self-renewal ability of CSCs derived from canine mammary cancer lines and focused on the candidate inhibitors targeting the mammalian target of rapamycin (mTOR) signaling pathway extracted by the *in vitro* screening.

## Materials and methods

### Cell lines and culture

The present study used two canine mammary carcinoma cell lines (CTBp and CNMp) (33). The cell lines were maintained in Dulbecco’s modified Eagle medium and nutrient F-12 (DMEM/F12, Invitrogen, Carlsbad, CA, USA), supplemented with 10% fetal bovine serum (Hyclone, Logan, UT, USA) and antibiotics (Nakarai Tesque, Kyoto, Japan) at 37°C in an atmosphere containing 5% CO_2_.

### Sphere-formation assay

The sphere-forming assay was performed as previously described (18). In brief, singly suspended cells derived from CTBp and CNMp were plated at a density of 1 × 10^3^ or 1 × 10^5^ viable cells per ultralow attachment 96-well plate (Coring, NY, USA) for drug sensitivity or 100-mm dish (Coring) for xenograft, respectively. The cells were grown in serum-free DMEM/F12 supplemented with 10 ng/mL of basic fibroblast growth factor (bFGF, Invitrogen, Carlsbad, CA, USA), 10 ng/mL of epidermal growth factor (EGF, Invitrogen), 4 mg/mL of heparin (Sigma-Aldrich, St. Louis, CA, USA), and NeuroBrew-21 (Miltenyi Biotech, Tokyo, Japan).

### *In vitro* drug screening and sensitivity assay

Sphere assay was performed using SCADS inhibitor Kit IV, including 39 molecule-targeted inhibitors, obtained from the Screening Committee of Anticancer Drugs, Japan (**Table 1**). Singly suspended cells were cultured in the presence of inhibitors (final concentration: 1 μM or 10 μM) from the beginning for 5 days. Everolimus (LC Laboratories, Boston, MA, USA) and temsirolimus (LC Laboratories) were used for inhibitor sensitivity assay of adherent cells and spheres derived from CTBp and CNMp. The adherent cells, seeded at 5 × 10^3^ cells/well on the 96-well plates, were cultured for 24 h and stimulated with a fresh culture medium containing seven different doses (final concentration: 0.0001, 0.001, 0.01, 0.1, 1, 10, or 100 μM) of everolimus or temsirolimus for 48 h. The sphere assay was performed under the same inhibitor concentration and using the *in vitro* screening protocol described above. Each living cell was evaluated using Cell Counting Kit-8 (Dojindo Laboratories, Kumamoto, Japan).

**Table 1:** The viability of the sphere-forming cells derived from canine mammary carcinoma cell lines cultured with various concentration of the inhibitors supplied in SCADS inhibitor kit IV.

SCADS kit4 inhibitors		Viability (% of control)			
		CTBp		CNMp	
Targets	Inhibitors	1μM	10μM	1μM	10μM
blank	none (DMSO)	100.00	100.00	100.00	100.00
Bcr-Abl	nilotinib	60.51	64.82	64.25	97.16
Multi-kinases	sorafenib	**12.85**	**11.21**	90.95	65.01
mTOR	temsirolimus	**27.64**	**28.30**	74.66	**29.27**
EGFR/Her2	lapatinib	**13.43**	**12.21**	63.35	**27.28**
Bcr-Abl/Kit	imatinib mesylate	136.30	139.07	68.33	105.99
Multi-kinases	sunitinib malate	**20.27**	**16.76**	139.37	108.65
EGFR	gefitinib	**12.47**	**10.77**	54.75	**20.55**
HDAC	vorinostat	**11.60**	**9.99**	60.63	**13.21**
EGFR	erlotinib	**13.22**	**12.10**	93.67	**22.01**
Proteasome	bortezomib	**13.26**	**11.88**	50.23	**12.01**
Bcr-Abl/Src	dasatinib	**13.22**	**11.88**	90.50	**32.82**
mTOR	everolimus	**31.16**	**30.41**	106.33	**46.29**
Multi-kinases	pazopanib	**13.76**	**12.76**	150.23	63.47
Rho/SRF	CCG-1423	67.68	57.60	106.79	121.89
PIM	PIM1/2 Kinase Inhibitor V	69.62	61.38	143.44	151.68
PIM	PIM1 Inhibitor II	101.99	85.68	113.57	100.97
Hedgehog	AY 9944	**12.76**	**11.43**	99.10	76.12
Hedgehog	cyclopamine	**44.63**	**36.85**	92.76	68.23
Hedgehog	Jervine	**47.20**	**39.29**	94.57	58.35
STAT3	WP1066	**13.39**	**11.99**	134.84	86.98
STAT3	5,15-DPP	67.55	59.49	143.89	128.26
Wnt	IWP-2	91.34	69.92	143.44	117.96
Wnt	IWR-1-endo	68.50	64.04	74.66	158.72
Wnt	FH535	72.81	68.92	75.11	109.54
Notch	DAPT	**47.16**	**38.07**	186.88	86.75
tankyrase-selective PARP	XAV939	112.14	89.57	145.70	178.41
pan-PARP	PJ-34	**41.07**	**30.74**	185.07	137.95
PARP-1/2-selective	Olaparib	**17.24**	**16.32**	115.38	92.66
antipsychotic drug	chlorpromazine hydrochloride	**25.90**	**43.51**	163.35	118.86
depression treatment	desipramine hydrochloride	103.44	80.36	208.14	128.52
golgi inhibitor	brefeldin A	**11.07**	**9.99**	176.02	109.02
stress inducer	anisomycin	**14.94**	**11.07**	**4.58**	**4.61**
thalidomide family	thalidomide	126.68	95.98	92.74	92.43
thalidomide family	lenalidomide	126.15	102.65	86.30	79.14
retinoids	tretinoin	94.34	78.28	66.90	**42.83**
retinoids	tamibarotene	106.01	109.54	**40.30**	51.73
DNA alkylation	temozolomide	132.52	103.36	66.54	86.24
EML4-ALK	crizotinib	**42.50**	**11.73**	**43.41**	**4.64**
mTOR	Torkinib	**36.08**	**12.11**	**17.45**	**6.15**

The viability of less than 50% are indicated by boldface. Data represet the mean of triplecate culture.

For analysis of mTOR signaling activity after inhibitor treatment, CTBp and CNMp cell lines were seeded at 5×10^4^ and 1×10^4^ cells in 35 mm dish for cell culture and 6-well plate for sphere-forming assay, respectively. Adherent cultures were replaced with medium containing everolimus or temsirolimus at a final concentration of 10 μM after 3 days of culture. In the sphere-forming assay, after culturing for 5 days, similar inhibitors were added at a final concentration of 10 μM. Cells were harvested 1 and 4 hours after the addition of the inhibitor, and western blotting described below was performed.

### Western blotting

The adherent and sphere-forming cells derived from CTBp and CNMp cells were collected by centrifugation and washed with phosphate-buffer saline. The cells were lysed in lysis buffer (Promega, Tokyo, Japan) with a protein inhibitor cocktail for 15 min. Approximately 10 μg of the extracted protein was analyzed with the following specific monoclonal antibodies against mTOR (clone 7C10, Cell signaling Technology, Tokyo, Japan), phospho-mTOR (Ser2448) (clone 49F9, Cell Signaling Technology), 4E-BP (clone 53H11, Cell Signaling Technology) and phospho-4E-BP (Thr37/46) (clone 236B4, Cell Signaling Technology), and polyclonal antibody against β-actin (Santa Cruz Biotechnology). The membranes were incubated with horseradish peroxidase-conjugated immunoglobulin G (IgG) (GE Healthcare, Tokyo, Japan). The immunoreactivity was detected using an ATTO EzWestLumi plus reagent (ATTO, Tokyo, Japan) and ImageQuant LAS4000 mini (GE Healthcare).

### Xenograft transplantation

Female BLAB/c nude mice, aged 8 weeks, were purchased from CLEA Inc. (Tokyo, Japan). A suspension of 1 × 10^6^ sphere-forming cells derived from CTBp was subcutaneously injected into the ventrolateral area under anesthesia. We administered saline (control, n = 6/group) or everolimus (Novartis Pharma, Basel, Switzerland, 4 mg/kg; oral n = 4/group) intraorally twice a week for 21 days after tumor formation was macroscopically confirmed. The tumor volume (V) was estimated using the following equation: V = [(length) × (width)^2^]/2. Experiments were approved by the Animal Experiments Committee of Nippon Veterinary and Life Science University and were performed following the Guidelines for Animal Experiments by the Nippon Veterinary and Life Science University.

### Histopathology

The tumors formed in nude mice were fixed with 10% neutral-buffered formalin and routinely embedded in paraffin wax for histological examination. Sections were stained with hematoxylin and eosin. Serial sections were immunostained using the streptavidin-biotin-peroxidase method with primary monoclonal antibodies specific for Ki67 (1:100, Dako, Denmark A/S, Glostrup, Denmark) and alpha-smooth muscle actin (SMA, 1:400, Dako), vascular endothelial growth factor (VEGF, 1:100, Santa Cruz Biotechnology, California, USA). Briefly, sections were treated in 0.03% H_2_O_2_ in 33% methanol at room temperature for 30 min for endogenous peroxidase blocking, following a pretreatment at 121°C for 20 min in citrate buffer (pH 6.0) for Ki67 and SMA, and at 121°C for 15min in citrate buffer (pH 9.0) for VEGF. The validation of antibodies was confirmed by a positive reaction with biopsy samples diagnosed with canine mammary adenocarcinoma or by a negative normal mouse IgG. The intratumor SMA-positive vessel and Ki67 index of tumor cell densities were evaluated as previously described (34). To evaluate the immunostaining intensity of VEGF, 5 high-power field (x400) of tumor tissue were selected and measured using Image J software.

### Statistical analysis

The results are presented as means ± standard deviation. Student *t*-test and Welch’s *t*-test were used for statistical analyses with R version 4.2.2. *P*-values of <0.05 was considered significant.

## Results

### *In vitro* library screening using molecular-targeted inhibitors in canine mammary adenocarcinoma cell lines

A sphere-formation assay was performed using a molecular-targeted inhibitor kit consisting of 39 types to extract inhibitors that suppress the self-renewal ability. A decreased value of ≥50% was found in 23 inhibitors in CTBp compared to control under conditions of final concentrations of 1 μM and 10 μM. Conversely, 4 and 12 inhibitors were extracted at final concentrations of 1 μM and 10 μM, respectively, in CNMp. Eleven inhibitors, such as EGF receptor (lapatinib, gefitinib), proteosome (bortezomib), stress inducer (anisomycin), and mTOR (temsirolimus, everolimus, and torkinib), were common between both lines (**Table 1**). This study focused on the mTOR signaling pathway evaluated by western blotting and the *in vitro* and *in vivo* antitumor effects of adherent cells and sphere-forming cells derived from canine mammary adenocarcinoma lines.

### Activated mTOR signal pathway was detected in both adherent and sphere-forming cells

**Figure 1:**
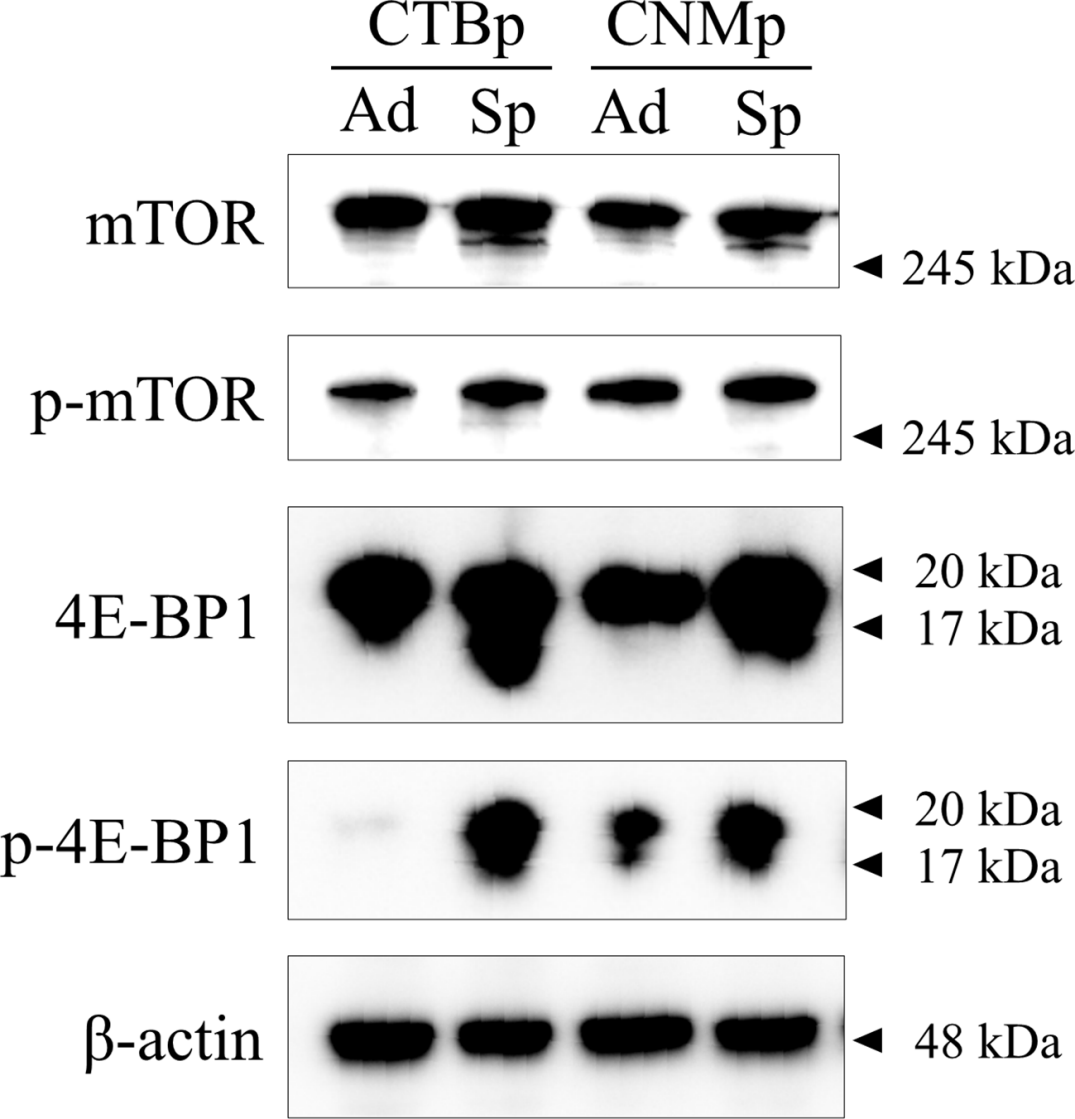
Western blot analysis of mTOR signaling in canine mammary adenocarcinoma lines, CTBp and CNMp. Adherent cells, ad; sphere-forming cells, Sp.

Western blotting was performed to confirm the expression of mTOR signal-related proteins, such as mTOR and 4E-BP1 in adherent and sphere-forming cells from canine mammary adenocarcinoma lines. Expression levels of mTOR and phosphorylated mTOR proteins were similar between adherent and sphere-forming cells of both lines (**Figure 1**). Conversely, 4E-BP1 was expressed in adherent and sphere-forming cells and phosphorylated 4E-BP1 was more highly expressed in sphere-forming than adherent cells of both lines (**Figure 1**). These results revealed that mTOR signaling was activated in both CTBp and CNMp.

### mTOR inhibitors, including everolimus and temsirolimus, inhibit adherent proliferation and sphere formation *in vitro*

**Figure 2:**
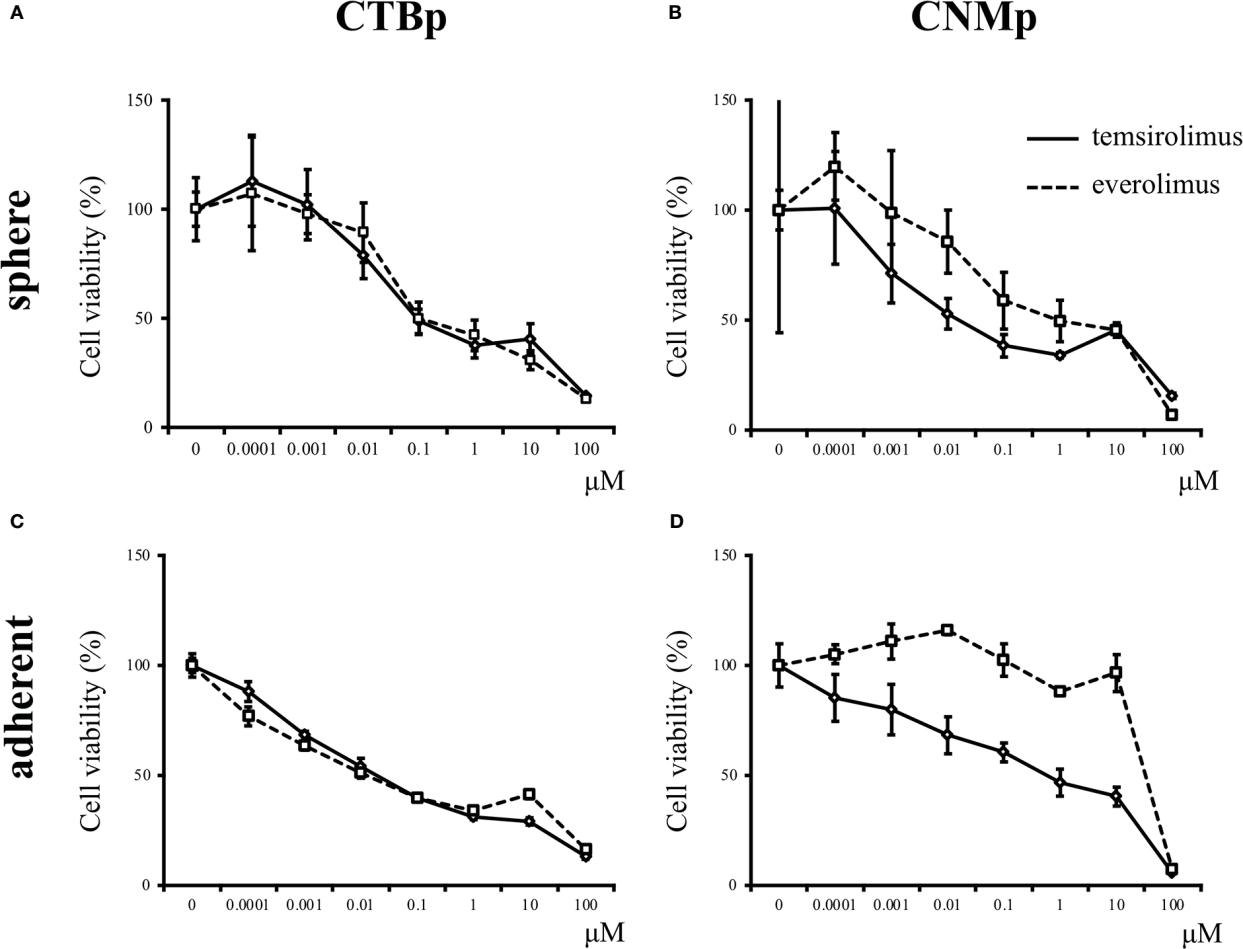
*In vitro* sensitivity assay of mTOR inhibitors, including everolimus and temsirolimus, in canine mammary adenocarcinoma lines, CTBp and CNMp. **(A, C)** CTBp and **(B, D)** CNMp. Upper shows for sphere-forming cells and lower shows the sensitivity assay results for adherent cells. The results shown are representative of at least three independent experiments.

*In vitro* sensitivity assay was performed using the mTOR inhibitors, including everolimus and temsirolimus, to examine inhibitory effects on sphere formation and adherent cell proliferation. Both CTBp and CNMp decreased the number of sphere-forming and adherent cells in a dose-dependent manner with everolimus and temsirolimus (**Figure 2**). The IC_50_ for everolimus and temsirolimus was 158.8 nM and 123 nM in CTBp-derived (**Figure 2A**) and 1.16 μM and 3.13 nM in CNMp-derived sphere-forming cells, respectively (**Figure 2B**). Whereas, that in CTBp- and CNMp-derived adherent cells were 17.0 nM and 39.5 nM (**Figure 2C**) and 53.6 μM and 52.9 nM (**Figure 2D**), respectively. In adherent cultures, the cell numbers of CTBp and CNMp lines were measured 24 and 48 hrs after treatment of inhibitors, and their numbers tended to be time-dependent (**Supplemental Figure 1**). Furthermore, in the sphere-forming assay, the number of CTBp-derived sphere-forming cells was measured 2 and 4 days after addition and was time-dependent similar to adherent cells (**Supplemental Figure 1**).

### mTOR inhibitors decrease the phosphorylation of mTOR signal in adherent and sphere-forming cells

**Figure 3:**
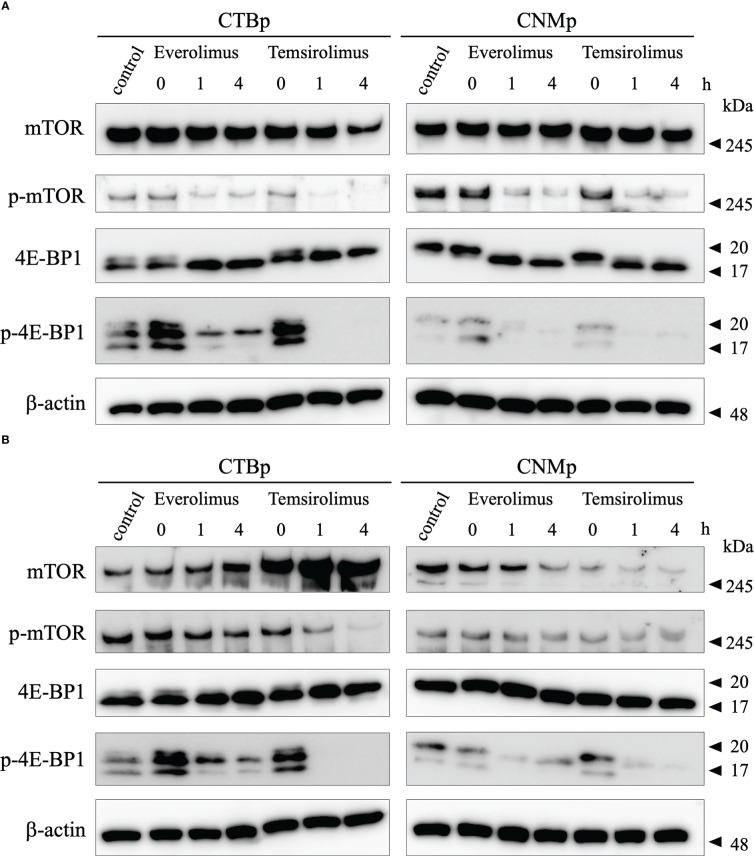
Western blot analysis of mTOR signal after evelorimus and temsirolimus treatment. **(A)** adherent cells, **(B)** sphere-forming cells.

Western blotting was performed to examine the expression of mTOR signal with 10 μM everolimus and temsirolimus treatment. In both cell lines, adherent cells decreased phosphorylated mTOR and 4E-BP1 expression 1 and 4 hours after inhibitor treatment (**Figure 3A**). On the other hand, sphere-forming cells treated with everolimus and temsirolimus also decreased phosphorylated 4E-BP1and mTOR expression (**Figure 3B**).

### Everolimus exhibits the antitumor effect in xenograft mice injected with CTBp-derived sphere-forming cells

**Figure 4:**
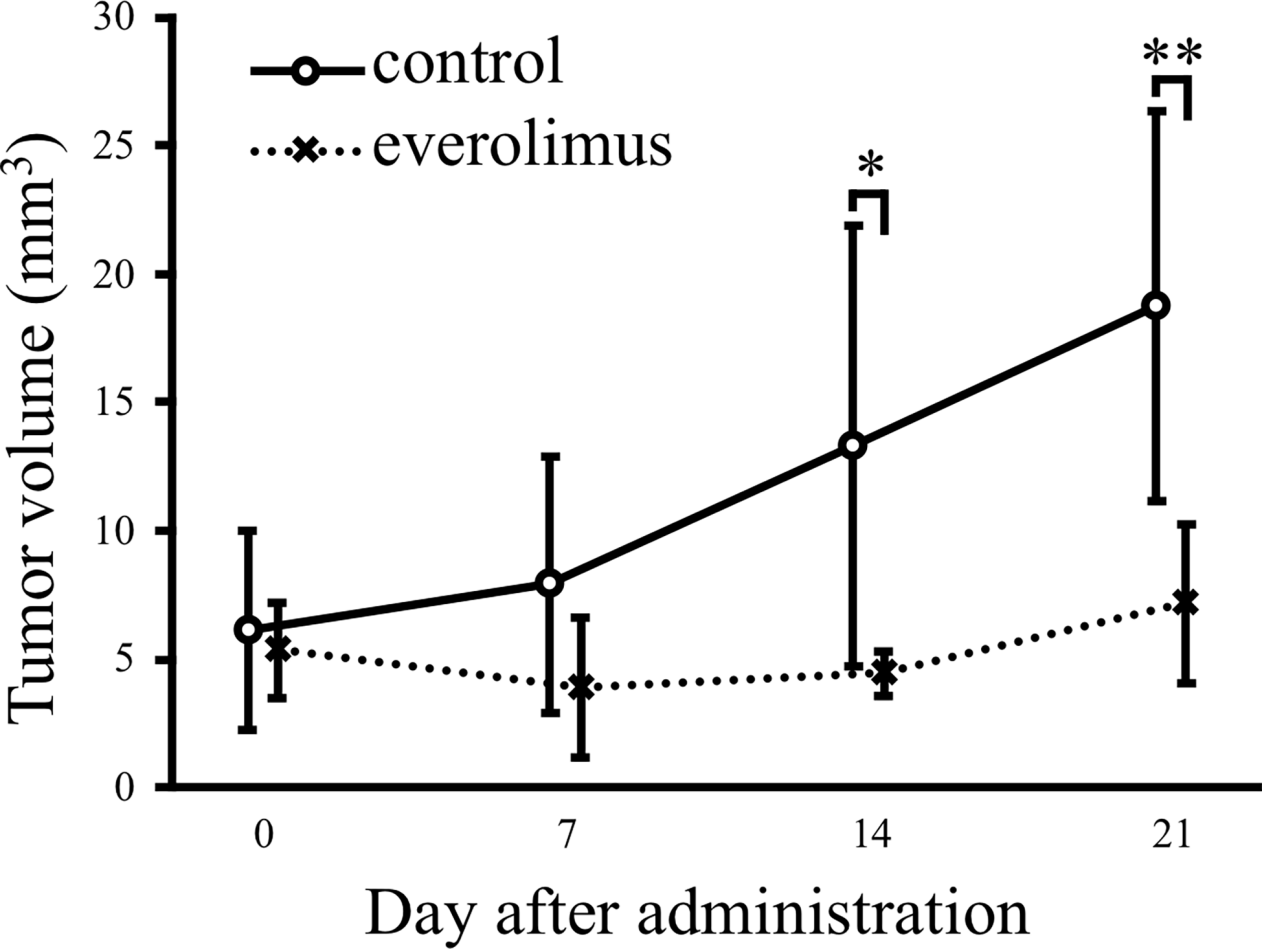
Antitumor effects of everolimus in a xenograft model transplanted canine mammary adenocarcinoma CTBp. Everolimus (n = 4, squares) or saline (n = 6, circles) was administrated twice per week for 21 days. The differences were tested by Scheffe’s F test. *P < 0.05, **P < 0.01.

**Figure 5:**
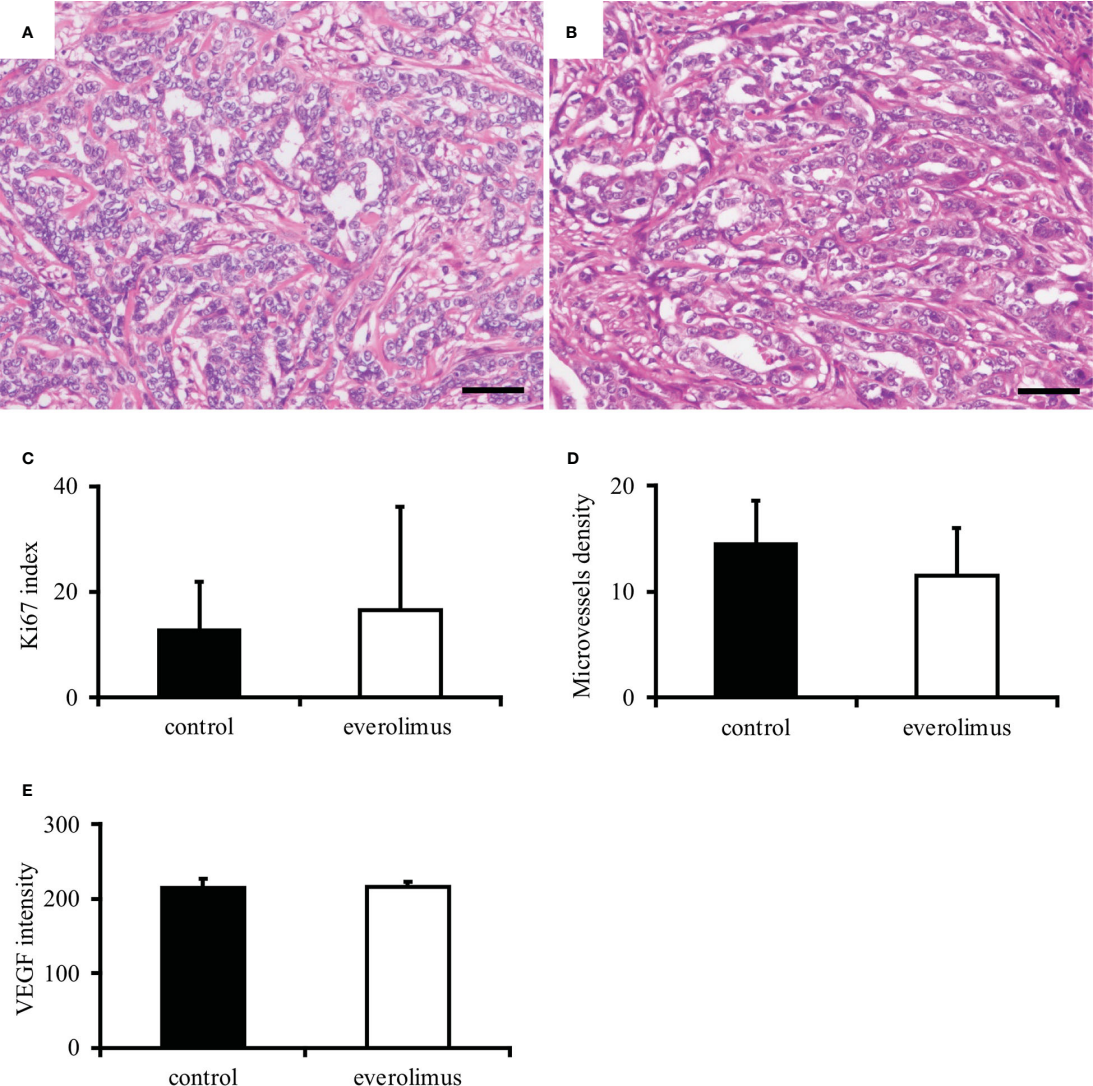
Histopathological evaluation of canine mammary carcinoma model mice. Tumors formed in xenografts show similar histology in both control **(A)** and everolimus-administered groups **(B)**. Hematoxylin and eosin. Scale bar = 50 μm. No significant difference was found in the Ki67 index **(C)**, smooth muscle actin-positive microvessels density **(D)**, and VEGF intensity **(E)** between the control and the everolimus-administered groups. The differences were determined by the Student *t*-test and Weltch’s *t*-test.

The *in vivo* antitumor effect of everolimus was investigated using mice transplanted with CTBp-derived sphere-forming cells. A significant tumor volume reduction was observed in the everolimus-administered group compared to the control group 14 and 21 days after administration (**Figure 4**). Histologically, the tumors formed in mice were similar in both groups and consisted of tubular or solid tumor cell proliferation (**Figures 5A, B**). Tumor necrosis and inflammatory cells, such as lymphocytes and mast cells, were not observed in both group. The Ki67 index of tumor cells was 12.72 ± 9.17 and 16.43 ± 19.69 in the control and everolimus-administered groups, respectively (**Figure 5C**). The number of intratumoral SMA-positive vessels was 14.48 ± 4.08 and 11.50 ± 4.51 in the control group and the everolimus-administered group, respectively (**Figure 5D**). Almost all tumor cells were positive for VEGF. VEGF immunostaining intensity of tumor cells was 214.5 ± 12.3 and 216.3 ± 6.49 in the control and evelorimus-administrated groups, respectively (**Figure 5E**). A significant difference was found in tumor volume, but with no significant difference between the two groups in both the Ki67 index, VEGF expression of tumor cells and the number of SMA-positive vessels.

## Discussion

This study conducted an *in vitro* library screening to suppress the self-renewal ability of spheres derived from canine mammary adenocarcinoma CTBp and CNMp lines and extracted molecular-targeted inhibitors, such as mTOR, hedgehog, and proteasome. This study focused on the mTOR signal. The activation of mTOR was observed in both sphere-forming cells and adherent cells of canine mammary cancer, and 4E-BP, which is an mTOR downstream signal, was activated in sphere-forming cells. Furthermore, *in vitro* sensitivity assay of everolimus and temsirolimus showed their growth suppression in both adherent cells and spheres, and everolimus revealed an antitumor effect in mice transplanted with sphere-forming cells. These results suggest that *in vitro* screening by sphere-formation assay using an inhibitor library is extremely useful for extracting inhibitors that suppress the self-renewal ability of CSCs in canine mammary carcinoma.

mTOR is a serine-threonine kinase that functions as a key downstream target of the phosphatidyl-inositol-3 kinase (PI3K)/AKT signaling pathway and has various regulatory functions, such as cell proliferation, metabolism, angiogenesis, and autophagy (35–37). mTOR forms a complex of mTORC1 and mTORC2, the 4E-BP1 and S6K exist downstream of mTORC1. Additionally, mTOR activation is associated with tumor development and plays an important role in maintaining the stemness of CSCs (36, 38). In human breast cancer, mTOR activation in CSCs is important for colony-forming and tumorigenicity (39). Activation of mTOR in CSCs has been reported in various cancers, such as colon cancer, prostate cancer, salivary gland cancer, and glioblastoma (40–43). mTOR signaling suppression reduces aldehyde dehydrogenase activity, which is abundant in immature cells, such as stem cells, in colon cancer (44). Therefore, mTOR signaling has attracted attention as a therapeutic target for various cancers (45). Everolimus has an inhibitory effect on breast CSCs (46, 47). Additionally, metformin exhibits antitumor effects on breast CSCs *via* AMP-activated protein kinase (AMPK) activation and mTOR suppression (48, 49). In dogs, mTOR phosphorylation has been detected by immunohistochemistry in various tumors, such as mammary tumors, squamous cell carcinoma, trichoblastoma, myxosarcoma, hemangiosarcoma, and prostate cancer (50–54). Phosphorylated mTOR expression in canine mammary tumors is associated with tumorigenesis and negative clinical behavior (51). Additionally, mTOR phosphorylation has been demonstrated in many cancer lines, such as osteosarcoma, melanoma, hemangiosarcoma, mast cell tumor, breast adenocarcinoma, glioma, and lymphoma, by western blotting, and rapamycin, which is an mTOR inhibitor, is associated with decreased mTOR phosphorylation and cell viability (50, 55–58). Cancer therapy targeting the PI3K/mTOR signaling pathway is expected to have an antitumor effect against canine mammary cancer and melanoma (59–61). The dual PI3K/mTOR inhibitor, VDC597, dose-dependently reduces cell proliferation, invasion, and vascular endothelial growth factor production in canine hemangiosarcoma (58). However, the role of mTOR signaling in canine mammary CSCs remains unclear. Therefore, this study proposes the use of mTOR inhibitors as targeted therapies for CSCs and cancer cells in canine mammary cancers, similar to human breast cancers. Unfortunately, the mechanism of the antitumor effect of everolimus in canine mammary cancer model mice could not be clarified because no difference was found in mitotic figures and angiogenesis between control and mTOR-administered groups. VEGF, which plays an important role in tumor angiogenesis, expression in tumor cells between control and everolimus-administrated groups supports the result that there is no difference in intratumoral angiogenesis between both group. Further studies will reveal the mechanisms underlying the antitumor effect of mTOR inhibitors.

Hedgehog (HH) signaling plays an important role not only in promoting embryonic development and cell differentiation but also in tumor initiation and progression (62). Additionally, HH signaling is essential not only for normal stem cells but also for maintaining CSC stemness (63). HH signaling pathway dysregulation in human breast cancer has been implicated in triple-negative and HER2-positive breast cancers and is persistently activated in CSCs, thereby promoting CSC’s self-renewal ability (63–66). Therefore, HH signaling is one of the cancer therapeutic targets. HH signaling in dogs is expressed in cancer cell lines, including osteosarcoma and transitional cell carcinoma, and HH inhibitors, such as cyclopamine, GANT61, and vismodegib, suppress tumor proliferation in these cancers (67–69). However, the antitumor effects in canine mammary cancer-containing CSCs remained unknown. In the present study, HH signaling, such as AY9944, cyclopamine, and jervine, is identified as a candidate to suppress the self-renewal ability of CSCs from the CTBp line, although detailed analysis has not been performed. Therefore, HH signaling may be a potential therapeutic target in canine mammary carcinoma, similar to human breast cancers.

The proteasome inhibitor, bortezomib, showed high sensitivity to canine mammary adenocarcinoma lines in this high-throughput screening. Bortezomib induces cell death *via* proteotoxic stress and alters the pro/anti-apoptotic protein balance by inhibiting ubiquitinated protein degradation by the 20S proteasome (70). Bortezomib is a Food and Drug Administration-approved therapeutic drug for multiple myeloma and mantle cell lymphoma (71, 72). Conversely, bortezomib monotherapy has had poor outcomes in patients with metastatic breast cancer, whereas a study reported 11 months of progression-free survival without adverse events in patients with metastatic triple-negative breast cancer with TP53 mutations (73, 74). CSCs are more resistant to bortezomib than differentiated cancer cells, but bortezomib-encapsulated nanoparticles can affect the stemness of CSCs compared to free bortezomib (75, 76). Veterinary medicine has shown higher sensitivity in canine cancer lines, including prostate cancer, lymphoma, melanoma, and osteosarcoma, as well as antitumor efficacy in melanoma-transplanted mice, but bortezomib sensitivity in CSCs has never been evaluated (77–80). Therefore, molecular-targeted therapy using bortezomib is expected to be beneficial as a cancer treatment for dogs. Further studies will reveal that bortezomib has antitumor effects in canine mammary cancer, although this study performed no detailed bortezomib analysis in canine mammary CSCs.

Sphere-forming cells are less sensitive to drugs, such as doxorubicin, carboplatin, and cyclooxygenase-2, than adherent cancer cells (20, 28, 29). *In vitro* library screening revealed the presence of inhibitors, including Wnt, PIM, and thalidomide family, that showed low sensitivity to the two concentrations used in this study, suggesting that all inhibitors are insentisitive to CSCs. Furthermore, further research is essential to determine whether inhibitors that are sensitive to sphere-forming cells can acquire resistance to them.

Sphere is a cancer stem cell population with self-renewal and differentiation ability (20). Characterization of sphere-forming cells in canine mammary carcinoma will lead not only to the elucidation of the pathogenesis of mammary carcinoma, but also to the development of therapies targeting CSCs (18). The tumor microenvironment plays a critical role in the stemness of CSCs, and also contributes to tumor progression and resistance to therapeutic agents (81, 82). The tumor microenvironment comprises a diverse population of cells, including fibroblasts, cancer-associated fibroblasts (CAFs), mesenchymal stem cells, endothelial cells, immune cells, such as T lymphocytes, macrophages, and dendritic cells (83). However, sphere-forming assay can not construct a microenvironment. Therefore, co-culture of spheres and CAFs can construct a microenvironment that is useful for further characterization of CSCs (84). Furthermore, organoids are formed in 3-dimentional cultures, but, unlike spheres, they form mimics of cancer tissues composed of CSCs, cancer cells, and microenvironment that are construct *in vivo* (85). In further research, in addition to spheres, *in vitro* drug screening targeting cancer organoids will be essential for the development of new therapeutic strategies in veterinary medicine.

Canine mammary cancer is an excellent spontaneous intermediate animal model for human breast cancer study, and new therapeutic studies for canine mammary cancer are a promising area in comparative oncology. However, the results of this study are limited to cell culture and mammary cancer model mice, and the therapeutic effect in dogs with mammary cancer remain unclear. In the future, clinical trials in dogs with breast cancer are essential. Therefore, establishing new therapeutic strategies and developing novel therapeutic protocols for canine mammary cancer is expected to bring beneficial benefits not only to veterinary medicine but also to human breast cancer treatment. Human and canine oncology need to collaborate in breast cancer research following the one health concept.

## Data availability statement

The original contributions presented in the study are included in the article/**Supplementary Material**. Further inquiries can be directed to the corresponding author.

## Ethics statement

The animal study was reviewed and approved by Animal Experimental Committee, Nippon Veterinary and Life Science University.

## Author contributions

MM: conception, design, and writing the draft. All authors contributed to data acquisition and analysis, and approved the submitted version.

## Supplementary material

Supplementary Figure 1 Time-susceptibility to everolimus and temsirolimus in mammary adenocarcinoma cells. CTBp **(A, B)** and CNMp **(C, D)** adherent cells treated with evelorimus and temsirolimus. CTBp-derived sphere-forming cells treated with evelorimus **(E)** and temsirolimus **(F)**.
