# Supplementary figures and images for: mTOR pathway as a potential therapeutic target for cancer stem cells in canine mammary carcinoma
Source: Front Oncol. 2023 Jan 27;13:1100602. doi: 10.3389/fonc.2023.1100602 (PMC9931192; doi:10.3389/fonc.2023.1100602)

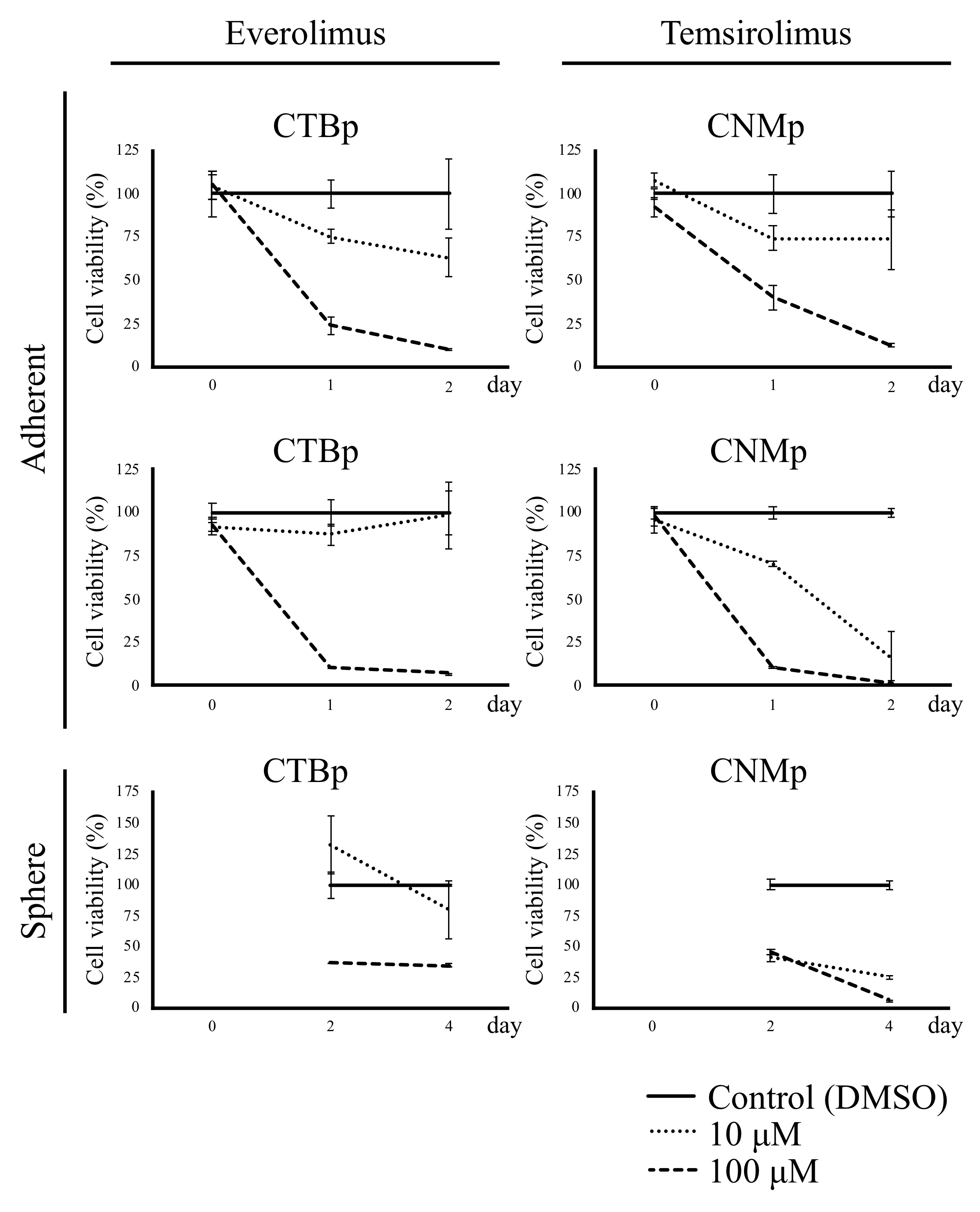

Supplement: Supplementary Figure 1 — Time-susceptibility to everolimus and temsirolimus in mammary adenocarcinoma cells. CTBp (A, B) and CNMp (C, D) adherent cells treated with evelorimus and temsirolimus. CTBp-derived sphere-forming cells treated with evelorimus (E) and temsirolimus (F). [file Image_1.tiff]
